# Trauma-Associated Pseudo-Hutchinson Sign: An Autobiographical Case Report Emphasizing Conditions, Pseudo-Conditions, and Pseudo-Pseudo-Conditions

**DOI:** 10.7759/cureus.76474

**Published:** 2024-12-27

**Authors:** Philip R Cohen

**Affiliations:** 1 Dermatology, University of California, Davis Medical Center, Sacramento, USA; 2 Medicine, Touro University California College of Osteopathic Medicine, Vallejo, USA; 3 Maples Center for Forensic Medicine, University of Florida College of Medicine - Gainsville, Gainsville, USA

**Keywords:** condition, hematoma, hutchinson, melanoma, nail, pseudo, sign, subungual, syndrome, trauma

## Abstract

Melanonychia describes black pigmentation of the nail plate that results from either melanocyte activation (such as infections, local inflammatory disorders, local trauma affecting the nail plate, numerous systemic conditions, and medications) or melanocyte hyperplasia (such as benign neoplasms or malignant tumors) or blood (resulting from a trauma-associated subungual hematoma). The black dyschromia may include not only the nail plate but also the proximal nailfold. The Hutchinson sign refers to black discoloration of both the proximal nailfold and adjacent nail plate when the underlying pigmented lesion is a malignant melanoma. The pseudo-Hutchinson sign describes these same morphologic features when the associated pigmented lesion is benign. The pseudo-pseudo-Hutchinson sign is used to describe the clinical scenario when there is non-black dyschromia of the proximal nailfold and nail plate, such as green caused by *Pseudomonas aeruginosa* infection or red resulting from an acute subungual hematoma. A 65-year-old man is described whose trauma-associated subungual hematoma of his left great toenail presented with a pseudo-Hutchinson sign. Sequential images of the distal toe, the proximal nailfold, and the nail plate were taken at two weeks, six weeks, 10 weeks, 14 weeks, and 18 weeks after the traumatic injury occurred. Two weeks after the injury occurred, the black discoloration of the pseudo-Hutchinson sign could be seen on the proximal nailfold and nail plate of the left great toenail; 16 weeks later, the pseudo-Hutchinson sign was not present. The black discoloration originally observed on the proximal nailfold had completely resolved; the black dyschromia of the proximal nail plate had progressed between 3 and 4 mm distally, and there was a normal-appearing nail plate on the left great toenail extending from the proximal nailfold. In addition to the Hutchinson sign, the pseudo-Hutchinson sign, and the pseudo-pseudo-Hutchinson sign, there are several conditions that are characterized by the condition, the pseudo-condition, and the pseudo-pseudo condition. The conditions include infections (such as bacterial), disorders (affecting the cardiovascular, endocrine, gastrointestinal, neurologic, ophthalmologic, or rheumatologic systems), and syndromes (that may or may not be associated with benign neoplasms or malignant tumors). In conclusion, a trauma-associated pseudo-Hutchinson sign of a 65-year-old man's left great toenail was described. The clinical observations confirmed the suspected diagnosis of the pseudo-Hutchinson sign. Additional evaluation, possibly including nail plate avulsion with an appropriate biopsy to determine the etiology of the pigmented lesion in the nail matrix and/or the nail bed, should be considered in an individual who develops features of the pseudo-Hutchinson sign that persist to exclude melanoma-associated Hutchinson sign.

## Introduction

Dyschromia of the nail plate can be associated with nail-related conditions or topical medications applied to the nail plate, dermatologic disorders, systemic diseases, and systemic drugs; the nail plate can be either black, blue, brown, green, red, white, or yellow. Black nail plates can be secondary to melanocytic lesions (such as benign neoplasms or malignant tumors) or non-melanocytic etiologies (such as fungal infections or trauma-associated subungual hematomas). If the proximal nailfold is black or brown, it is either referred to as the Hutchinson sign (when the underlying pigmented lesion is a malignant melanoma) or the pseudo-Hutchinson sign (when the associated pigmented lesion is benign). However, when the pigmentation of the proximal nailfold and the nail pigmentation is neither black nor brown, it is designated as a pseudo-pseudo-Hutchinson sign [1-3].

A 65-year-old man is described who developed a trauma-associated pseudo-Hutchinson sign of his left great toenail. The black subungual pigmentation appeared not only beneath his toenail but also on his proximal nailfold. The nail plate dyschromia progressed distally as the nail plate continued to grow and the discoloration of the proximal nailfold spontaneously resolved.

Like the pseudo-pseudo-Hutchinson sign, there are several conditions that are characterized by the condition, the pseudo-condition, and the pseudo-pseudo-condition. An example is hypoparathyroidism, pseudohypoparathyroidism, and pseudo-pseudo hypoparathyroidism [4,5]. Signs and syndromes characterized by the condition, the pseudo-condition, and the pseudo-pseudo-condition are summarized [1-20].

## Case presentation

A 65-year-old man dropped a small plastic container from a height of three feet; the container fell on the distal phalanx of his left great toe. His past medical history was significant for an atrial flutter-associated cerebrovascular accident, and he was on 5 mg of apixaban twice daily. The site of impact was extremely painful, yet there was no clinical change immediately following the incident.

**Figure 1:**
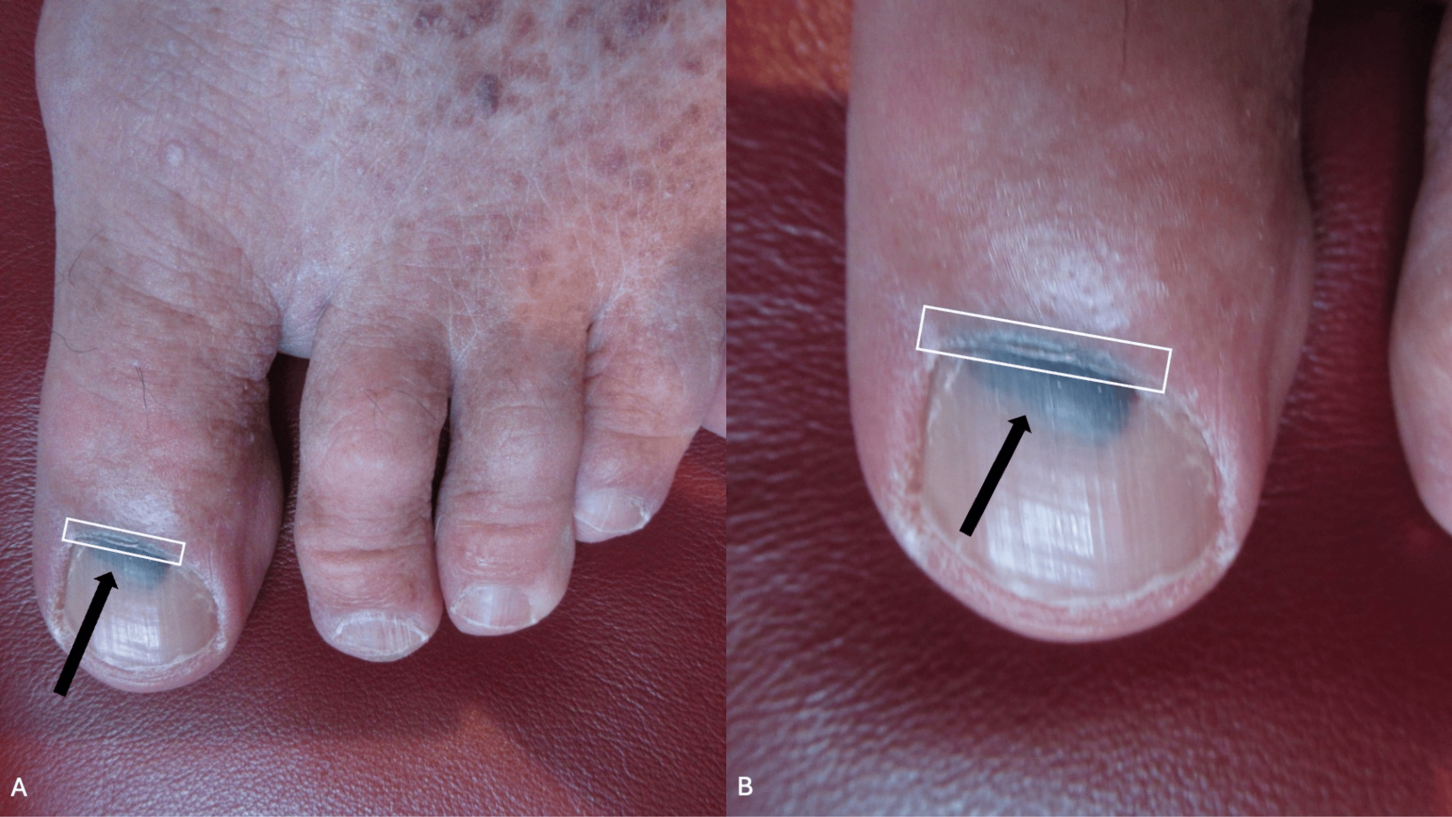
Trauma-associated pseudo-Hutchinson sign on the left great toenail A distant view (A) and a closer view (B) of the black dyschromia of the left great toenail and its proximal nailfold can be observed two weeks following the trauma to the distal left great toe. Black discoloration appeared after a plastic container dropped on the distal toe of a 65-year-old man who received 5 mg of the oral anticoagulant apixaban twice daily. The proximal nailfold (outlined by the white rectangle) shows black discoloration, and the proximal nail plate exhibits black subungual dyschromia (black arrow).

Within two weeks, a black discoloration had appeared beneath the proximal left great toenail; in addition, the proximal nailfold was also black (Figure 1). The clinical differential diagnosis included Hutchinson sign (a subungual pigmented lesion associated with black discoloration of the proximal nailfold, which is associated with malignant melanoma) and pseudo-Hutchinson sign (non-melanoma-related black dyschromia of both the nail plate and proximal nailfold). Based on the history of recent trauma to the digit and the acute onset of the lesion, the diagnosis of pseudo-Hutchinson sign caused by a subungual hematoma was favored.

**Figure 2:**
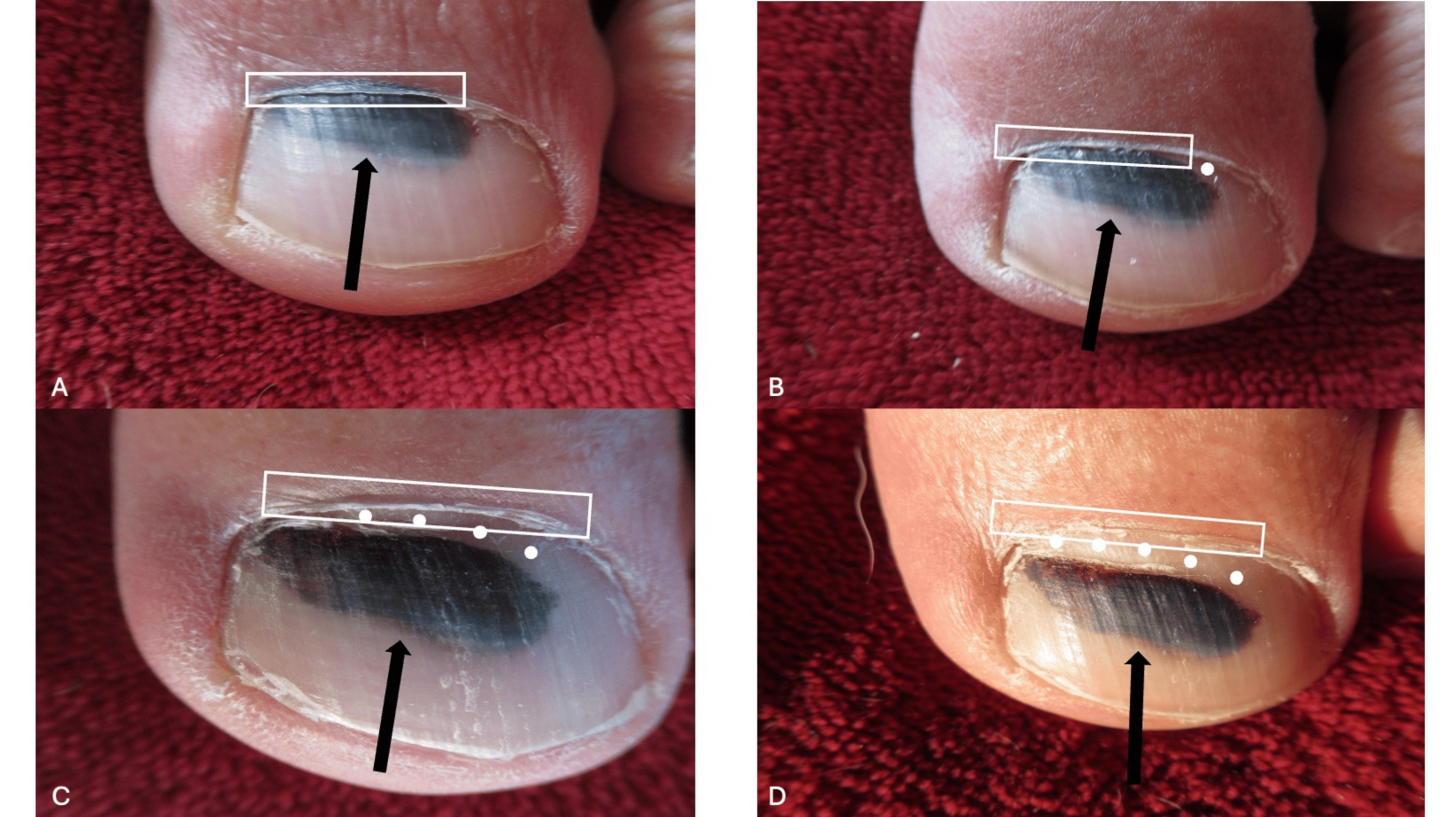
Sequential views of the left great toenail of a 65-year-old man The distal left great toe after blunt trauma from a plastic container. Images of the distal toe, the proximal nailfold, and the nail plate were taken at six weeks (A), 10 weeks (B), 14 weeks (C), and 18 weeks (D) after the injury occurred. The white rectangle outlines the proximal nailfold and the black arrow points to the subungual pigmentation of the nail plate. The solid white circles demonstrate the normal-appearing growth of the proximal nail plate; toenails typically grow at a rate of 1 mm per month. The black dyschromia on the proximal nailfold (A and B), along with black discoloration of the contiguous nail plate, demonstrating a positive pseudo-Hutchinson sign, can be observed. At 10 weeks following the traumatic event (B), there is 1 mm of normal proximal nail plate growth. At 14 weeks (C) and 18 weeks (D) post-trauma, the normal proximal nail plate is 2-3 mm and 3-4 mm, respectively. The proximal nailfold is completely without any black dyschromia at 18 weeks (D).

The left great toenail was monitored clinically. Each month, photographs were performed (Figure 2). The sequential images demonstrated that, eventually, the black discoloration of the proximal nailfold spontaneously resolved.

**Figure 3:**
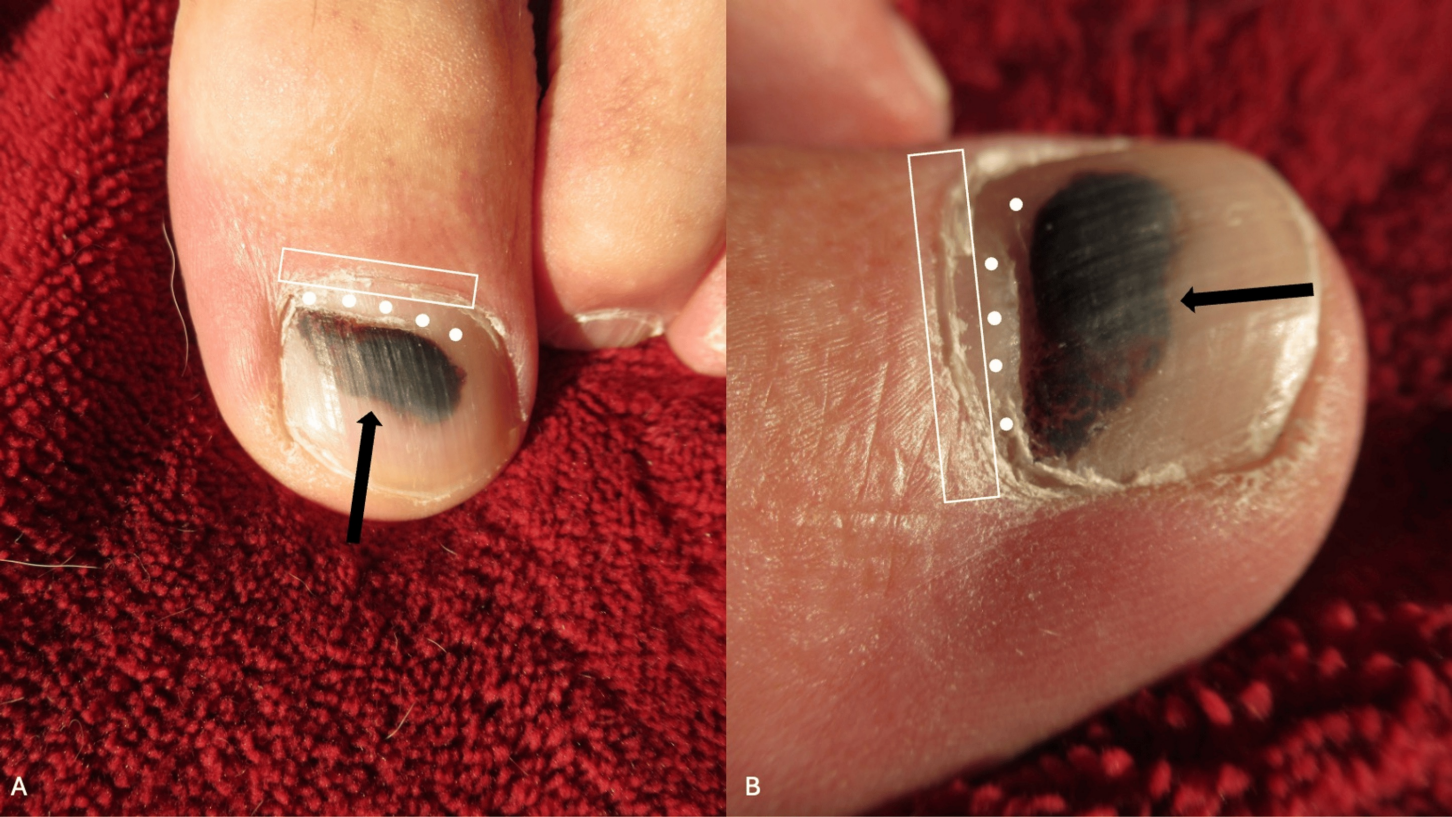
Resolution of the pseudo-Hutchinson sign Distant (and frontal) view (A) and closer (and lateral) view (B) of the left great toe of a 65-year-old man at 18 weeks following the trauma to the digit. The solid white circles demonstrate the normal-appearing growth of the proximal nail plate. The black arrow points to the subungual pigmentation of the nail plate. The white rectangle outlines the proximal nailfold. The black discoloration has not only completely resolved from the proximal nailfold but also from the proximal left great toenail; 3-4 mm of the normal-appearing nail plate is present between the proximal nailfold and the residual black subungual hematoma.

Eighteen weeks following the trauma to the distal left great toe, all the black dyschromia was gone from the proximal nailfold (Figure 3). Normal-appearing growth of the proximal left great toenail (measuring 3-4 mm) was observed.

**Figure 4:**
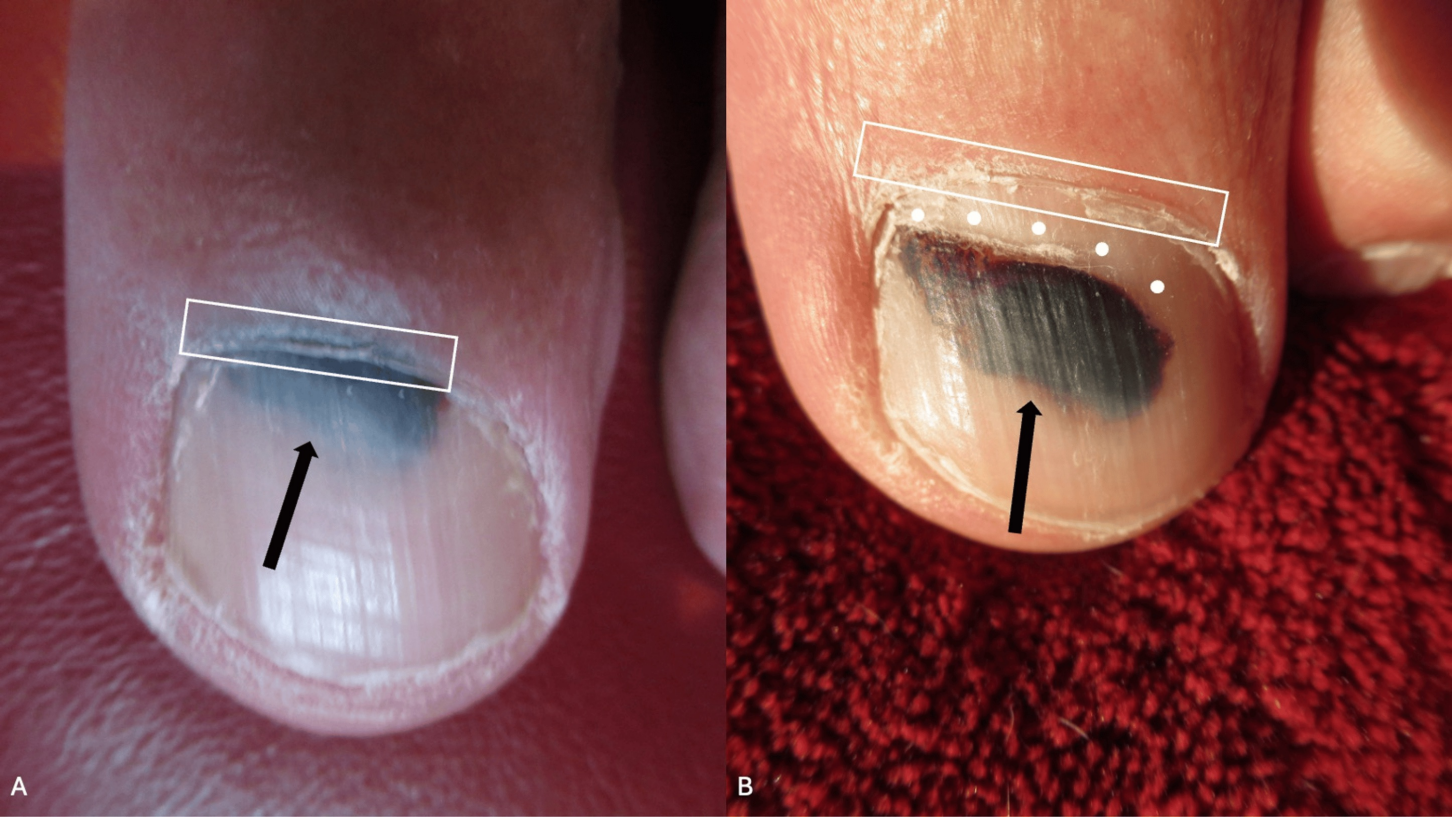
Distal left great toe: comparison between the presentation of the pseudo-Hutchinson sign and the subsequent spontaneous resolution of the pseudo-Hutchinson sign Two weeks after trauma (A), the black discoloration of the pseudo-Hutchinson sign can be seen on the proximal nailfold and nail plate of the left great toenail; 16 weeks later (B), the pseudo-Hutchinson sign is not present. The proximal nailfold is outlined by the white rectangle, and the black arrow shows the subungual black pigmentation; the normal-appearing growth of the proximal nail plate is demonstrated by the solid white circles. The black discoloration originally observed on the proximal nailfold has completely resolved; the black dyschromia of the proximal nail plate has progressed 3-4 mm distally, and there is a normal-appearing nail plate on the left great toenail extending from the proximal nailfold. The residual trauma-induced black discoloration, resulting from the subungual hematoma, will continue to progress distally as the nail plate grows.

The correlation of the history of trauma to the affected distal toe, the clinical presentation of the lesion, and the subsequent resolution of the nailfold discoloration in concert with the appearance of normal-appearing proximal nail growth and distal progression of the nail plate dyschromia toward the free edge of the nail plate established the diagnosis of pseudo-Hutchinson sign associated with trauma-related subungual hematoma (Figure 4).

## Discussion

Melanonychia refers to the black pigmentation of the nail plate. It can be caused by melanocyte activation, melanocyte hyperplasia, or blood (from trauma to the nail). Melanocyte activation includes infections, local inflammatory disorders or trauma affecting the nail plate, numerous systemic conditions, and medications. Melanocyte hyperplasia includes pigmented lesions of the nail bed, such as lentigos, melanocytic nevi, and melanoma [1-3]. The black nail plate was caused by blood (a subungual hematoma) in the reported patient.

The patient in this report initially not only had melanonychia but also a black proximal nailfold. Initially, the morphologic presentation was compatible with both the Hutchinson and pseudo-Hutchinson signs. The history was consistent with a pseudo-Hutchinson sign: an abrupt onset of nailfold and nail plate black dyschromia following a traumatic injury to the area. The growth rate of toenails is approximately 1 mm a month; after several months, the suspected diagnosis of pseudo-Hutchinson's sign associated with a subungual hematoma was established by observing spontaneous resolution of the nailfold black discoloration and growth of a normal-appearing nail proximal to the melanonychia.

The Hutchinson sign and the fiber sign both have a pseudo-sign and a pseudo-pseudo sign [1-3,6-8]. Similarly, several conditions are characterized by not only the condition but also the pseudo-condition and the pseudo-pseudo-condition (Table 1) [1-20]. The conditions include infections (such as bacterial), disorders (affecting the cardiovascular, endocrine, gastrointestinal, neurologic, ophthalmologic, or rheumatologic systems), and syndromes (that may or may not be associated with benign neoplasms or malignant tumors) [4,5,9-20].

**Table 1:** Conditions and signs, pseudo-conditions and pseudo-signs, and pseudo-pseudo-conditions and pseudo-pseudo-signs CA-125: cancer antigen-125; LUPLE: lupus-associated protein-losing enteropathy; Ref: references

Conditions and Signs	Definition	Ref
Bacteremia	Bacteremia is the presence of bacteria in a patient’s bloodstream.	[9]
Pseudo-bacteremia	Pseudo-bacteremia is a false-positive blood culture result. The cause of pseudo-bacteremia can be breaks in technique while the blood culture was being obtained or processed. In addition, pseudo-bacteremia can be caused by exposure to contaminated solutions, devices, or glassware during the blood culture process.	[9,10]
Pseudo-pseudo-bacteremia	Pseudo-pseudo-bacteremia was reported in 12 patients who had blood cultures on the same day and received reports in their hospital charts of positive blood culture results for Neisseria gonorrhea; none of the patients had a clinically compatible syndrome. However, all the blood culture results in the laboratory had been reported as negative on the original worksheets. Investigation revealed that a positive cervical culture for Neisseria gonorrhea had been printed out by the computer just before printing out the 12 erroneous blood culture reports. Indeed, the computer printout error was the result of a subtle mistake in the computer program. In summary, this series of cases of pseudo-pseudo-bacteremia (false-positive culture results) was caused by a computer; the computer error was corrected, and amended reports were sent.	[9]
Dementia	Dementia is a chronic disease that causes symptoms affecting memory, thinking, and social abilities; there is a decline in cognitive abilities (such as remembering, reasoning, and thinking) that interferes with daily life.	[11]
Pseudo-dementia	A syndrome in which dementia is mimicked or caricatured by functional psychiatric disorders is pseudo-dementia.	[11]
Pseudo-pseudo-dementia	When pseudo-dementia is simulated by diseases other than psychiatric disorders (such as organic brain disease), it is termed pseudo-pseudo-dementia.	[11]
Fiber sign	The fiber sign is a dermatoscopic clue to ulceration of a skin lesion; it occurs since serum leaking from ulcerated areas may trap fibers of clothing or loose hair. Ulceration is most commonly seen in basal cell carcinoma; importantly, a biopsy should be performed when ulceration (without a clear history of trauma) is observed, either clinically or dermatoscopically, in a skin lesion.	[6]
Pseudo-fiber sign	The pseudo-fiber sign is a novel dermoscopic sign of nail psoriasis; it was described as red and black filamentous structures located along the cuticle or underneath the distal free edge on the hyponychium or exposed areas where the nail plate had detached. Because these structures closely resemble the dermoscopically visible adherent fibers that result from lesion excoriation or ulceration, the researchers designated the sign as the pseudo-fiber sign.	[7,8]
Pseudo-pseudo-fiber sign	The pseudo-pseudo-fiber sign was reported by other investigators (Lencastre et al.) of nail psoriasis after they closely evaluated the published images of the pseudo-fiber sign described by Yorulmaz and Artuz. Lencastre et al. noted that one of the figures clearly showed that a red ‘pseudo-pseudo-fiber’ jumps over the proximal/lateral nailfold onto the nail; they postulate that the patient may have been wearing wool mittens. In summary, the later researchers describe the pseudo-pseudo-fiber sign as merely artefactual and created by real fibers caught on a dystrophic nail.	[7,8]
Foster Kennedy syndrome	Foster Kennedy syndrome consists of the triad of ipsilateral optic atrophy, contralateral optic disc edema, and ipsilateral anosmia. The initial six patients described by Foster Kennedy in 2011 had expanding frontal lobe lesions, demonstrating ipsilateral optic atrophy with contralateral papilledema; ipsilateral anosmia was added in 1916.	[12,13]
Pseudo-Foster Kennedy syndrome	Pseudo-Foster Kennedy syndrome is characterized by non-compressive and non-tumor causes. The most common cause of pseudo-Foster Kennedy syndrome is anterior ischemic optic neuropathy; other non-tumor causes include occult trauma, optic neuritis, syphilis, and severe arteriosclerosis of the internal carotid arteries causing compression of the optic nerves by the carotid arteries.	[12,13]
Pseudo-pseudo-Foster Kennedy syndrome	Pseudo-pseudo-Foster Kennedy syndrome is two diseases (ischemic optic neuropathy and meningioma) in the same patient. An 80-year-old woman presented with anterior ischemic optic neuropathy in her left eye. Slow and progressive decreased vision (ostensibly secondary to a cataract) developed in her right eye. The slight temporal pallor of the optic disc was demonstrated in the right eye, and a superior temporal field defect was found. A tuberculum sella meningioma extending into the right optic canal, compressing the right optic nerve, was demonstrated on a radiologic exam.	[12,13]
Hutchinson sign	The Hutchinson sign is melanoma-associated black or brown pigmentation involving both the nail plate and the proximal nailfold.	[1,2]
Pseudo-Hutchinson sign	The pseudo-Hutchinson sign is black or brown pigmentation that involves the nail plate and the proximal nailfold; it is associated with non-melanoma lesions. The lesions occur because of either melanocyte activation (ethnic pigmentation, malnutrition, minocycline use, radiation therapy), melanocyte hyperplasia (lentigo, melanocytic nevus), infections (dermatophyte or yeast), syndromes (Laugier-Hunziker syndrome or Peutz-Jegher syndrome) or trauma (subungual hematoma).	[2,3]
Pseudo-pseudo-Hutchinson sign	The pseudo-pseudo-Hutchinson sign results from pigmentation that affects not only the nail plate but also the proximal nailfold, which is not brown or black in color; the discoloration can be green (when caused by a *Pseudomonas aeruginosa* bacterial infection) or red (when it is caused by an acute subungual hematoma).	[2]
Hypoparathyroidism	Hypoparathyroidism is characterized by low parathyroid, low calcium, and high phosphate serum levels. Primary (idiopathic) hypoparathyroidism results from failure of the parathyroid gland to secrete parathyroid hormone. Secondary hypoparathyroidism (characterized by a low or absent parathyroid level) has numerous causes: neck surgery (for cancer, goiter, nodules, hyperthyroidism), radiation therapy to the neck, magnesium depletion, autoimmune diseases, and inherited conditions. The appearance of affected individuals is normal.	[4]
Pseudo-hypoparathyroidism	Pseudo-hypoparathyroidism is due to the deficient end-organ response to circulating parathyroid hormone; it is characterized by high parathyroid hormone, low calcium, and high phosphate serum levels. Four types exist: type Ia, type Ib, type Ic, and type II. Type 1a and type 1c have a phenotypic expression of Albright hereditary osteodystrophy: short stature, below normal intelligence, obesity, round face, short neck, subcutaneous ossifications, brachydactyly, and shortened metatarsals. Type 1a and type 1b are caused by a gene defect (loss of function mutation in GNAS) inherited from the mother.	[4,5]
Pseudo-pseudo-hypoparathyroidism	Pseudo-pseudo-hypoparathyroidism has the phenotypic appearance of pseudo-hypoparathyroidism type Ia with normal labs; the parathyroid hormone, calcium, and phosphate serum levels are all normal. The gene defect is inherited from the father, and affected individuals do not have parathyroid hormone resistance.	[4,5]
Meigs syndrome	When Meigs syndrome was originally described in 1937, it was characterized by ovarian fibroma, ascites, and hydrothorax; CA-125 testing did not exist. Some of the patients with Meigs syndrome have elevated serum and/or ascitic CA-125. Subsequently, the syndrome was defined as a benign ovarian tumor (such as Brenner tumors, fibromas, fibrothecomas, granulosa cell tumors, sclerosing stromal tumors, and thecomas), ascites, and pleural effusion; following removal of the tumor, the ascites and pleural effusion swiftly resolve.	[14]
Pseudo-Meigs syndrome	Pseudo-Meigs syndrome is characterized by hydrothorax and ascites associated with pelvic masses; the patients occasionally present with elevated serum CA-125 levels. The syndrome-related tumors are conditions other than those observed in Meigs syndrome; they include ovarian malignancies (primary or metastatic ovarian tumors), ovarian struma ovarii, uterine cancers, uterine hemangioma, uterine leiomyoma, and yolk sac tumors.	[14]
Pseudo-pseudo-Meigs syndrome	Pseudo-pseudo-Meigs syndrome, also referred to as Tjalma syndrome, is characterized by massive ascites, pleural effusion, and significantly elevated serum CA-125 levels in patients with systemic lupus erythematosus, in the absence of an ovarian tumor. There are several conditions that can result in elevated serum CA-125 levels, including LUPLE, lupus peritonitis, nephrotic syndrome, and pelvic infections such as tuberculosis. However, to establish the diagnosis of pseudo-pseudo-Meigs syndrome, conditions that can cause polyserositis (such as lupus peritonitis or lupus nephritis with nephrotic syndrome) must be excluded.	[14]
Myocardial infarction	A decreased or complete cessation of blood flow, caused by an occlusion of one or multiple coronary arteries, to a portion of the myocardium, can cause a myocardial infarction. The evaluation of a myocardial infarction includes clinical factors, electrocardiogram findings, and cardiac biomarkers. Electrocardiograms in an acute myocardial infarction may include hyperacute T-waves or ST-segment changes (often elevation) or both.	[15]
Pseudo-myocardial infarction	A pseudo-myocardial infarction can be attributed to conditions that have electrocardiogram changes that resemble those observed in an acute myocardial infarction. Some of the conditions that can present as a pseudo-myocardial infarction are acute pancreatitis, pulmonary emphysema, pneumothorax, pulmonary embolism, hypertrophic cardiomyopathy, myocardial fibrosis, pheochromocytoma, intracranial hemorrhage, hyperkalemia, and acute pericarditis.	[15]
Pseudo-pseudo-myocardial infarction	A pseudo-pseudo-myocardial infarction results when the electrocardiogram demonstrates changes consistent with those observed in an acute myocardial infarction because of an artifact. An example of such an artifact would be a motion of the electrode or its connection, such as an arterial pulse wave altering skin contact with the arm lead with each heartbeat. Another example would be misplacement of the leads, resulting in a 'myocardial infarction simulated from improper telemetry' which is also referred to as a 'MISFIT' since there is a 'mis'diagnosis of a myocardial infarction that does not 'fit' with the clinical scenario.	[15,16]
Obstruction	Intestinal obstruction is a partial or complete blockage of the small intestine or large intestine that prevents food or liquid from passing. Symptoms include abdominal pain, cramps, vomiting, and/or the inability to pass feces or gas. The diagnosis can be suggested by clinical examination; hyperactive, tinkling, metallic, or high-pitched bowel sounds can be heard on stethoscope examination. Abdominal radiologic studies can also be helpful, including roentgenograms, computerized tomography, ultrasound, and/or air or barium enema.	[17,18]
Pseudo-obstruction	Chronic intestinal pseudo-obstruction is a rare condition characterized by symptoms and signs of intestinal obstruction; however, the lumen of the intestines is not obstructed by a mechanical lesion and the intestines fail to propel their contents. The condition may be idiopathic or caused by other diseases (such as amyloidosis or scleroderma), visceral neuropathies (involving the intrinsic enteric nervous system), or visceral myopathies (involving the enteric smooth muscle). Deranged smooth-muscle alpha-actin has been proposed to be a biomarker of the condition. Individuals with this condition often undergo one or more exploratory laparotomies before the diagnosis is established.	[17,18]
Pseudo-pseudo-obstruction	Pseudo-pseudo-obstruction is the occurrence of actual mechanical intestinal obstruction occurring in an individual who has an established diagnosis of chronic intestinal pseudo-obstruction based on clinical, radiological, and histopathological features. Adhesions may develop in patients with chronic intestinal pseudo-obstruction; thereby, a previously absent obstruction may develop. In one woman, clues to the diagnosis included small intestine manometric features (suggesting a distal mechanical obstruction) and worsening of clinical symptoms after initiating treatment with a prokinetic agent (cisapride).	[17,18]
Seizure	A seizure is a sudden change in behavior, movement, or consciousness caused by abnormal electrical activity in the brain. The uncontrolled burst of electrical activity between the brain cells causes temporary abnormalities in muscle tone (limpness, stiffness, or twitching), behaviors, sensations, or states of awareness.	[19,20]
Pseudo-seizure	Pseudo-seizure refers to non-epileptic seizure-like events unaccompanied by acute cerebral dysfunction nor by paroxysmal epileptiform electrical activity. Pseudo-seizure represents a psychiatric disease; convulsive pseudo-seizure is the most encountered.	[19]
Pseudo-pseudo-seizure	Pseudo-pseudo-seizure is the occurrence of epileptic seizures in individuals who are initially misdiagnosed as having a psychiatric illness.	[20]

The difference between the sign or the condition and the pseudo-sign or the pseudo-condition may include the clinical presentation, the associated features, and/or the biochemical characteristics. Similarly, the pseudo-pseudo-sign or the pseudo-pseudo-condition may include the morphology, the related characteristics, and/or the laboratory features [1-20]. The appearance of the nail plate and proximal nailfold of the patient in this report were compatible with both Hutchinson sign and pseudo-Hutchinson sign; however, the spontaneous resolution of the proximal nailfold black dyschromia and concurrent growth of normal proximal nail plate provided morphologic confirmation of the correct diagnosis of pseudo-Hutchinson sign [1-3].

## Conclusions

The Hutchinson sign is caused by subungual malignant melanoma; there is black pigmentation of the nail plate (melanonychia) and the adjacent proximal nailfold. The same morphologic features, associated with a benign etiology, are referred to as a pseudo-Hutchinson sign. Non-black dyschromia of the proximal nailfold and nail plate is designated as a pseudo-pseudo-Hutchinson sign. There are numerous conditions (including infections, systemic disorders, and syndromes) that are characterized by the condition, the pseudo-condition, and the pseudo-pseudo-condition; several are summarized in this report.

A 65-year-old man with black discoloration of the proximal nailfold and adjacent nail plate of his left great toe was described. The clinical differential diagnosis included the Hutchinson sign and trauma-associated pseudo-Hutchinson sign. However, based on the history of recent trauma to the digit and the acute onset of the lesion, the diagnosis of pseudo-Hutchinson sign caused by a subungual hematoma was favored. Follow-up evaluations of the black proximal nailfold and adjacent subungual pigmentation demonstrated that the discoloration of the proximal nailfold spontaneously resolved, and the nail plate dyschromia progressed distally as the nail plate continued to grow. Additional evaluation, possibly including nail plate avulsion with an appropriate biopsy to determine the etiology of the pigmented lesion in the nail matrix and/or the nail bed, should be considered in an individual who develops features of the pseudo-Hutchinson sign that persist to exclude melanoma-associated Hutchinson sign. The black discoloration initially observed on the proximal nailfold of the patient's left great toe completely resolved within 18 weeks after the traumatic injury occurred. In addition, 3-4 mm of normal-colored nail plate appeared on the left great toenail between the proximal nailfold and the residual black dyschromia on the nail plate. In conclusion, these findings confirmed the suspected diagnosis of pseudo-Hutchinson sign associated with a subungual hematoma.
